# Roles of neurally adjusted ventilatory assist in improving gas exchange in a severe acute respiratory distress syndrome patient after weaning from extracorporeal membrane oxygenation: a case report

**DOI:** 10.1186/s40560-016-0153-4

**Published:** 2016-04-07

**Authors:** Yuya Goto, Shinshu Katayama, Atsuko Shono, Yosuke Mori, Yuya Miyazaki, Yoko Sato, Makoto Ozaki, Toru Kotani

**Affiliations:** Department of Anesthesiology and Intensive Care Medicine, Tokyo Women’s Medical University, Tokyo, 162-8666 Japan; Department of Anesthesiology, Shimane University, Shimane, 693-8501 Japan

**Keywords:** Neurally adjusted ventilatory assist, Compliance, Patient-ventilator synchrony, Electromyography, Electrical impedance tomography, Ventilation distribution

## Abstract

**Background:**

Patient-ventilator asynchrony is a major cause of difficult weaning from mechanical ventilation. Neurally adjusted ventilatory assist (NAVA) is reported useful to improve the synchrony in patients with sustained low lung compliance. However, the role of NAVA has not been fully investigated.

**Case presentation:**

The patient was a 63-year-old Japanese man with acute respiratory distress syndrome secondary to respiratory infection. He was treated with extracorporeal membrane oxygenation for 7 days and survived. Dynamic compliance at withdrawal of extracorporeal membrane oxygenation decreased to 20 ml/cmH_2_O or less, but gas exchange was maintained by full support with assist/control mode. However, weaning from mechanical ventilation using a flow trigger failed repeatedly because of patient-ventilator asynchrony with hypercapnic acidosis during partial ventilator support despite using different types of ventilators and different trigger levels. Weaning using NAVA restored the regular respiration and stable and normal acid-base balance. Electromyographic analysis of the diaphragm clearly showed improved triggering of both the start and the end of spontaneous inspiration. Regional ventilation monitoring using electrical impedance tomography showed an increase in tidal volume and a ventilation shift to the dorsal regions during NAVA, indicating that NAVA could deliver gas flow to the dorsal regions to adjust for the magnitude of diaphragmatic excursion. NAVA was applied for 31 days, followed by partial ventilatory support with a conventional flow trigger. The patient was discharged from the intensive care unit on day 110 and has recovered enough to be able to live without a ventilatory support for 5 h per day.

**Conclusion:**

Our experience showed that NAVA improved not only patient-ventilator synchrony but also regional ventilation distribution in an acute respiratory distress patient with sustained low lung compliance.

## Background

Initiation and termination of inspiratory support during conventional partial ventilatory support is triggered by changes in flow (flow trigger, FT) or pressure (pressure trigger) in the airway. Trigger delay can cause late initiation and early termination of mechanical support [1]. This is known as patient-ventilator asynchrony, leading to not only insufficient inspiratory assist but also irregular, disturbed respiration. Asynchrony is more obvious and often a problem when the patient is converted to partial ventilatory support for the weaning from mechanical ventilation and gas exchange is insufficiently maintained, although it is observed during full ventilatory support (e.g., assist/control ventilation). Asynchrony exaggerates pre-existing acute respiratory failure [2]. Neurally adjusted ventilatory assist (NAVA) uses the electromyographic activity of the diaphragm (EAdi) detected through an electrode placed in the stomach. NAVA triggers the initiation and termination of ventilatory support and improves patient-ventilator synchrony [3–5].

We experienced a case of severe acute respiratory distress syndrome (ARDS) in which initiating NAVA markedly improved the patient’s respiratory status. In this case report, we investigated the roles of NAVA in improving respiratory status.

## Case presentation

**Fig. 1:**
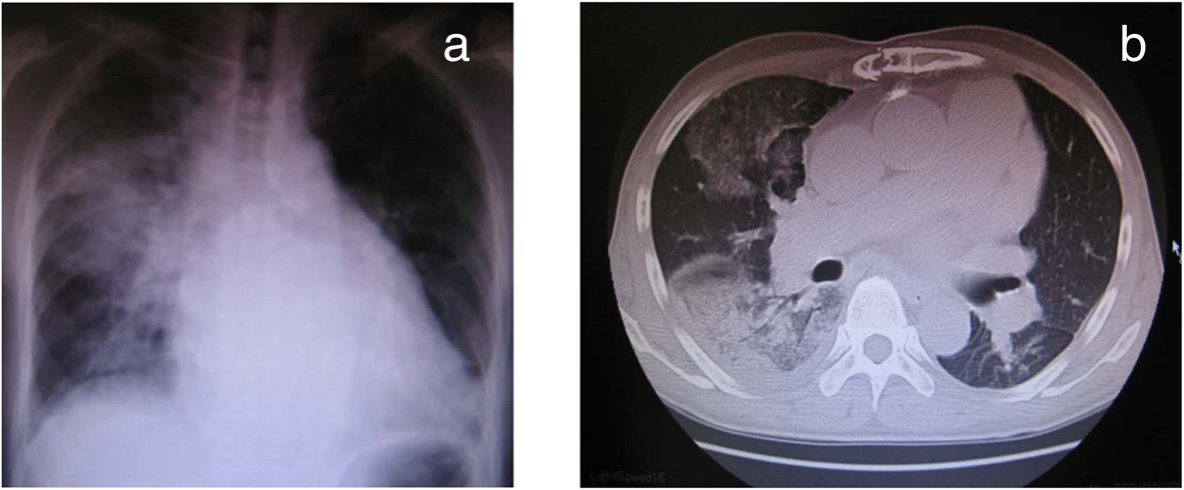
Chest radiograph (**a**) and chest computed tomography (**b**) at hospital admission

**Fig. 2:**
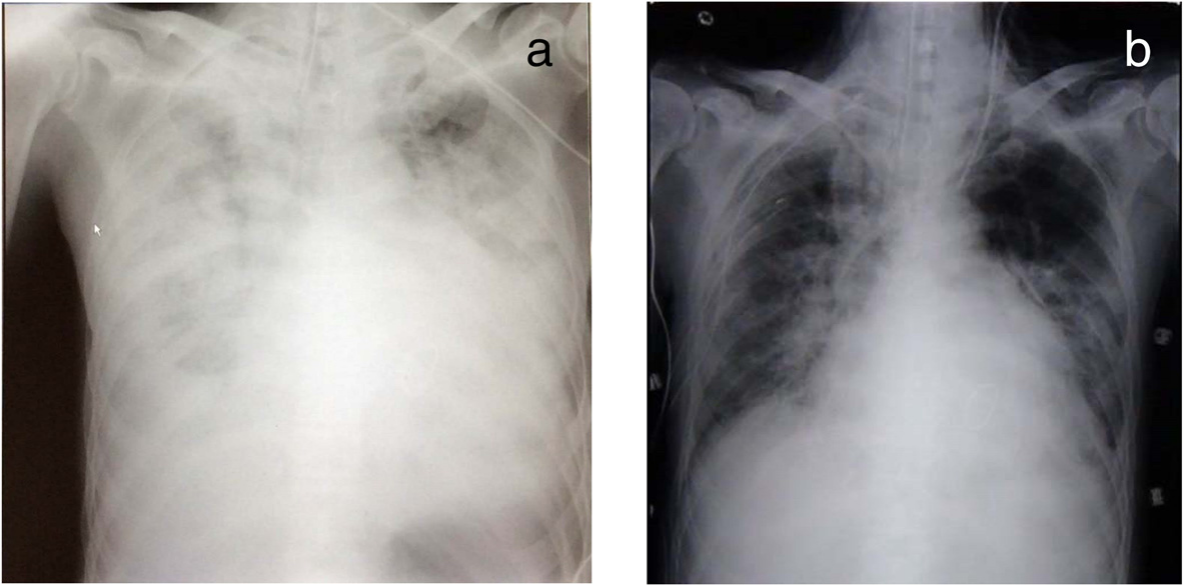
Chest radiographs at ICU admission (**a**) and at weaning from ECMO (**b**)

The patient was a 63-year-old Japanese man with a history of mitral valve plasty. He presented with general fatigue and cough of 1 month’s duration with worsening symptoms. When he presented to the respiratory medicine clinic, he had a fever of 39 °C and infiltration was observed in the right lung field on chest radiographs (Fig. 1). He was suspected of having bacterial pneumonia and was admitted. Although antibiotic therapy with ampicillin/sulbactam and minomycin was started, he presented with acute respiratory failure on day 3; therefore, noninvasive positive pressure ventilation and a new antibiotic regimen (piperacillin/tazobactam and ceftazidime) were started. Peramivir was also started because rapid diagnostic test for influenza-B appeared positive. Microbial testing could not detect the cause of pneumonia. Pulse steroid therapy was then initiated at 1 g/day on days 4–6 and days 16–18 and was followed by daily steroid therapy at 60 mg/day to treat the respiratory failure. On day 18, the patient developed pneumomediastinum and pneumothorax and because acute respiratory distress syndrome developed after chest drainage, the patient was transferred to our intensive care unit (ICU) on day 20 (Fig. 2a). He was immediately intubated and mechanically ventilated. Arterial blood gas analysis revealed pH, 7.131; PaCO_2_, 76.2 mmHg; PaO_2_/FIO_2_ (P/F), 86.9 mmHg; and HCO_3_^−^, 20.9 mmoL/L. Gas exchange could not be improved with any ventilatory modality or setting; therefore, veno-venous extracorporeal membrane oxygenation (V-V ECMO) with a pump flow of 3 L/min was established using a 23-Fr internal jugular drainage cannula and a 19-Fr femoral return cannula. As the chest radiographic findings improved and the oxygenation during a minimal pump flow test met the weaning criteria for ECMO [6] 7 days later (day 26), the patient was successfully weaned from V-V ECMO (Fig. 2b). Dynamic compliance at that time decreased to 20 ml/cmH_2_O or less, but gas exchange remained stable using an assist/control mode (inspiratory pressure 26 cmH_2_O, inspiratory time 0.8 s, positive end-expiratory pressure [PEEP] 7 cmH_2_O). However, tachypnea (>40 breaths/min) and frequent reflective cough were observed when the weaning process was initiated with a change to synchronized intermittent mandatory ventilation (SIMV; inspiratory pressure of 26 cmH_2_O and inspiratory time of 0.8 s for mandatory ventilation, PEEP 7cmH_2_O) and pressure support ventilation (PSV; pressure support 18 cmH_2_O above PEEP with expiration cycling time 25 %). The patient became anxious and restless, and his respiration was irregular and unstable, resulted in respiratory acidosis (pH, 7.215; PaCO_2_, 58.9 mmHg; P/F, 220 mmHg; and HCO_3_^−^, 20.8 mmoL/L). Although we adjusted various trigger levels and pressure support levels with several different types of ventilators and different ventilation modalities (two automatic weaning systems and proportional assist ventilation), adequate gas exchange could not be maintained and the ventilation mode was returned to assist/control soon after the weaning trial performed on three consecutive days. Tracheostomy was performed 2 days after weaning from ECMO.

**Fig. 3:**
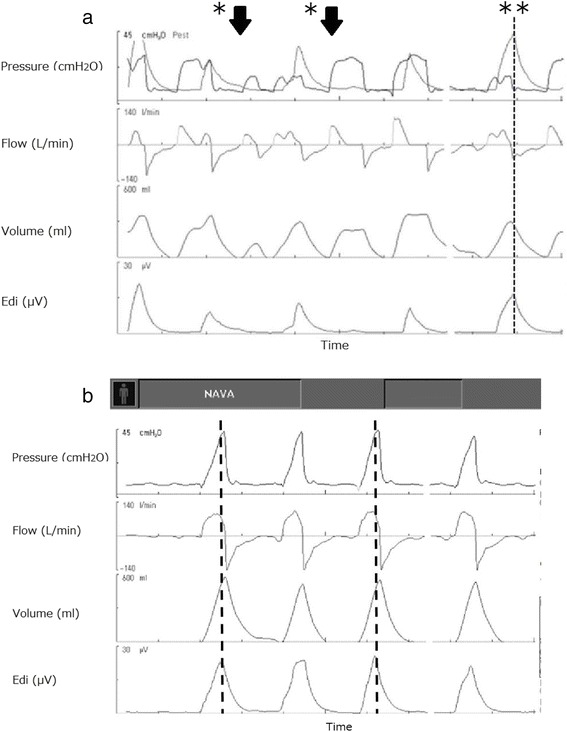
Ventilator graphics during flow trigger (**a**) and NAVA (**b**). These two figures were obtained just after conversion from assist/control mode. *Top* pressure-time curve, *mid-top* flow-time curve, *mid-bottom* tidal volume-time curve, *bottom* electromyography of diaphragm. *Dashed line* termination of inspiration guided by electromyography of diaphragm. Improved synchrony after initiating NAVA is obvious compared with SIMV. *NAVA* neurally adjusted ventilatory assist, *SIMV* synchronized intermittent mandatory ventilation. *Single asterisk* autotriggering, *double asterisks* premature cycling

It was obvious that patient-ventilator asynchrony was the major cause of the weaning failure. To improve synchrony, we introduced NAVA because of the completely different triggering system from FT. After implementing NAVA, the incidence of reflective cough decreased. Respiratory rate and tidal volume in SIMV and NAVA were 50/min and 30/min and 250–300 and 450–550 ml, respectively, with the same PEEP. The patient communicated improved comfort, and we were able to continue the weaning process. Concurrent analysis of electromyography of the diaphragm and airway pressure and inspiratory flow waveforms clearly demonstrated auto triggering, trigger delay for the diaphragm contraction, and premature cycling in FT (Fig. 3a). In contrast, no signs of asynchrony were observed during the use of NAVA. Initiation and termination of inspiration were well synchronized, which decreased the respiratory rate from 48 to 30 breaths/min and increased the minute volume (Fig. 3b). EAdi was 12–20 μV in SIMV and increased to 25–40 μV after initiating NAVA.

**Fig. 4:**
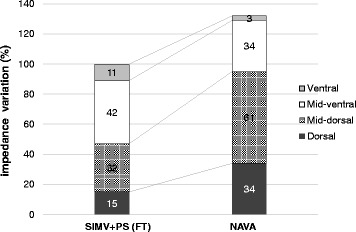
Changes in regional ventilation and global tidal impedance measured by electrical impedance tomography. Figure shows values during flow trigger (*FT*, *left*. PSV 18 cmH_2_O and expiration cycling time 25 %, PEEP 7 cmH_2_O) and *NAVA* (*right*. Gain 1 cmH_2_O/μV, PEEP 7 cmH_2_O). *Numbers on the bar* indicate the proportion of ventilation distribution in each lung region. Global tidal impedance increased 1.32 times after NAVA. *NAVA* neurally adjusted ventilatory assist, *PEEP* positive end-expiratory pressure

**Fig. 5:**
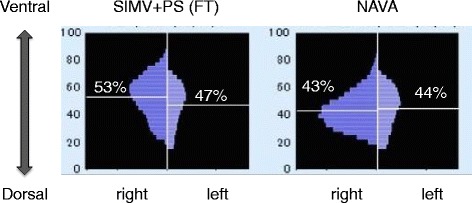
Center of ventilation (COV) in each lung calculated by electrical impedance tomography. Figure shows values during flow trigger (*FT*, *right*) and *NAVA* (*left*). The sum of ventilation-related impedance change in each lung slice is calculated and presented as a *bar* in the histograms. *Horizontal lines* indicate the position of COV. COV shifted to the dorsal region during NAVA in the right lung. *FT* flow trigger, *NAVA* neurally adjusted ventilatory assist, *COV* center of ventilation

Regional ventilation was assessed using electrical impedance tomography (EIT) when the ventilation mode was changed from assist/control mode. NAVA increased the distribution of ventilation in the mid-dorsal and dorsal regions from 32 and 15 to 46 and 26 %, respectively. Global tidal impedance variation, which reflects tidal volume, was 1.32 times higher with NAVA (gain 1 cmH_2_O/μV, PEEP 7 cmH_2_O) compared with SIMV (Fig. 4). The center of ventilation also shifted to the dorsal region (from 53 to 43 % in the right lung and from 47 to 44 % in the left lung) during NAVA (Fig. 5).

Peak value of EAdi gradually decreased to 10–20 μV at which level NAVA was successfully converted to conventional PSV of 24 cmH_2_O 31 days later. The patient was discharged from our ICU on day 110 with a home ventilator to enhance his mobility program. As of day 325, the patient has recovered enough to be able to live without a ventilator support 5 h per day.

### Discussion

While the number of survivors following ECMO increases, we have also seen more cases of difficult weaning from mechanical ventilation in patients whose oxygenation is restored and relatively stable; some appear to have sustained low lung compliance. Patient-ventilator asynchrony is a major reason for difficult weaning from mechanical ventilation [7], and low lung compliance can cause asynchrony.

During conventional PSV, patients can both trigger and cycle the breath. However, mechanical ventilation using FT is not initiated until airway flow generated by diaphragmatic contraction reaches a pre-determined level. Trigger delay is often observed in patients with low lung compliance. Because of the physical properties of the respiratory system, negative pressure generated by inspiratory muscle contraction is difficult to transmit to the airway flow change. Longer delays between respiratory muscle contraction and actual gas flow generation cause patient-ventilator asynchrony and patient discomfort. Also, with low lung compliance, the inspiratory flow decreases rapidly after the peak and reaches the termination threshold of mechanical support very early. The result is that flow support using FT is initiated with a delay and terminated earlier despite the fact that diaphragmatic contraction continues. These effects result in serious shortening of the duty cycle, increased patient work of breathing, patient-ventilator asynchrony, and the inability to adjust inspiratory effort [8–12].

Theoretically, NAVA has potential in these patients because it is immediately triggered by the electrical signal from spontaneous diaphragmatic contraction and decreases asynchrony. However, there are few reports of its use. In our case, the clinical symptom showed that FT caused asynchrony; the patient had excessive inspiratory effort and tachypnea because of low lung compliance. Asynchrony exaggerated tachypnea, caused cough, and increased work of breathing. These exhausted the patient and finally led to a cycle of respiratory failure. Another reason for asynchrony was the difference of supporting pressure. Assist/control mode maintained gas exchange, and the patient’s inspiratory effort was overridden. However, conversion from assist/control to SIMV caused vigorous inspiration effort due to insufficient support. Supporting pressure of NAVA was set automatically corresponding to the patient’s inspiratory effort (electrical activity of diaphragm) and was higher than that of PSV. The flow waveform of PSV is a decelerating, triangular pattern (Fig. 3a), while that of NAVA is sinusoidal shape (Fig. 3b) that is identical to the shape of the muscle tension generated by the diaphragm, suggesting the better synchrony during NAVA. Patient-ventilator synchrony improved considerably after the introduction of NAVA, as shown in Fig. 2. More importantly, the improved synchrony decreased the work of breathing and supported the weaning process.

Patients with low lung compliance are considered candidate for NAVA when conventional partial ventilatory support fails to synchronize and worsens respiratory failure. The cost and reliability of long-term use of an EAdi catheter are limitation in the clinical application of NAVA.

A previous study reported the effect of NAVA in difficult weaning cases. Mauri and colleagues evaluated and compared asynchronies between PSV and NAVA in ARDS patients with low static compliance (18 ± 8 mL/cmH_2_O) undergoing ECMO. The authors found that the incidence of premature cycling, ineffective triggering, double triggering, and auto-triggering decreased [13]. In our case, auto triggering and premature cycling disappeared after initiating NAVA. NAVA broke the cycle of respiratory failure and immediately decreased the respiratory rate.

EIT is a clinically available noninvasive technique to assess global and regional ventilation distribution as well as end-expiratory lung volume at the patient’s bedside. Regional ventilation monitoring using EIT provided important findings in our case. The center of ventilation describes how ventilation is distributed between ventral and dorsal lung regions, and the value is less than 50 % in the supine position in spontaneously breathing human subjects. PSV to achieve sufficient tidal volume induces the distribution of ventilation toward the ventral region [14], especially when applying higher pressure, probably because patient effort is outpaced by applied flow and pressure development [15]. However, in our patient, we saw a dorsal shift of ventilation with the higher supporting pressure during NAVA, compared with PSV (Figs. 3 and 5). A previous study reported that NAVA reduced overassistance and had a beneficial effect on the ventilation of dependent lung region [16].

EAdi did not decrease after NAVA and this finding is compatible with previous studies [17, 18], although another study reported a decrease [19]. Previous studies reported that control mechanical ventilation is potentially sufficient to decrease diaphragm efficiency [20, 21]. PSV often overassists the movement of the diaphragm, whereas NAVA automatically adjusts the level of pressure support to the magnitude of inspiration effort, and reduces the risk of overassistance due to downregulation of the EAdi signal and improved diaphragm efficiency [22]. In our case, the strong inspiratory effort sustained for days after the conversion to NAVA probably due to the low lung compliance. NAVA, however, could maintain the support level not to be excessive and adjust for the magnitude of diaphragmatic excursion, resulted in the ventilation distribution predominant to the dependent lung regions.

## Conclusion

The findings in our case suggests that NAVA is worth trying for weaning to improve not only patient-ventilator synchrony but also regional distribution in an ARDS patient with sustained low lung compliance after weaning from ECMO.

### Ethics approval

The study was approved by our institutional ethics committee (reference number 3013).

### Consent

Written informed consent was obtained from the patient's kin for publication of this case report. A copy of the written consent is available for re- view by the Editor-in-Chief of this journal. 

## Abbreviations

EAdi: electromyographic activity of the diaphragm
ECMO: extracorporeal membrane oxygenation
EIT: electrical impedance tomography
FT: flow trigger
ICU: intensive care unit
NAVA: neurally adjusted ventilator assist
PEEP: positive end-expiratory pressure
PSV: pressure support ventilation
SIMV: synchronized intermittent mandatory ventilation
